# Tendon Dysfunction in Collagen VI-Related Myopathies: Novel Mechanistic Insights with Therapeutic Potential

**DOI:** 10.3390/ijms262412014

**Published:** 2025-12-13

**Authors:** Patrizia Sabatelli, Alberto Di Martino, Cesare Faldini, Paolo Bonaldo, Luciano Merlini, Vittoria Cenni

**Affiliations:** 1 CNR-Institute of Molecular Genetics, 40136 Bologna, Italy; 2 IRCCS, Istituto Ortopedico Rizzoli, 40136 Bologna, Italy; 3 1st Orthopedics and Traumatology Department, IRCCS Istituto Ortopedico Rizzoli, 40136 Bologna, Italy; albertocorrado.dimartino@ior.it (A.D.M.); cesare.faldini@ior.it (C.F.); 4 Department of Biomedical and Neuromotor Science, DIBINEM, University of Bologna, 40136 Bologna, Italy; mrllcn@unife.it; 5 Department of Molecular Medicine, University of Padova, 35122 Padova, Italy; bonaldo@bio.unipd.it

**Keywords:** COL6-related myopathies, Ullrich congenital muscular dystrophy, collagen VI, CMD, tendon extracellular matrix, tendon dysfunction, mechanotransduction, joint contractures, primary cilium, focal adhesion

## Abstract

Collagen VI-related myopathies (COL6-RM) encompass a spectrum of disorders characterized by muscle weakness, joint contractures, and connective tissue abnormalities resulting from mutations in the collagen VI genes. While muscle pathology has been extensively studied, tendon dysfunction has emerged as a critical yet underexplored contributor to disease severity, particularly in the development of joint contractures. Tendons from patients and animal models show disrupted collagen fibrillogenesis, altered extracellular matrix (ECM) composition, and impaired cellular mechanotransduction. Various defects in ECM remodeling pathways further exacerbate tendon pathology. Importantly, current clinical management remains limited to orthopedic interventions with modest outcomes, and targeted pharmacological strategies or gene-editing therapies are not yet available for clinical application. Therefore, understanding the basic pathogenic mechanisms underlying tendon dysfunction is essential for identifying novel therapeutic targets. This review provides a comprehensive synthesis of current understanding and recent advances concerning the role of mutated collagen VI in cellular and molecular mechanisms underlying tendon dysfunction. Emphasis is placed on the role of mutated collagen VI in the modulation of key signaling pathways related to mechanotransduction and primary cilium function in COL6-RM. By discussing these multifaceted contributions to disease pathogenesis, this review outlines future research directions in the field and highlights potential pathways for targeted therapeutic interventions.

## 1. Introduction

Collagen VI is a microfibrillar collagen expressed in the stroma of most tissues, including muscle, tendon, ligaments, and skin [1]. It forms a network of microfilaments connected with both ECM ligands and cell surface receptors. Collagen VI assembly and secretion are complex processes involving intracellular and extracellular steps. In humans, five genes, namely *COL6A1*, *COL6A2*, *COL6A3*, *COL6A5*, and *COL6A6*, encode five distinct α-chains, which intracellularly form triple-helical heterotrimeric monomers. Mutations in *COL6A1*, *COL6A2*, and *COL6A3* are causative for a heterogeneous group of disorders collectively known as COL6-RM. Specifically, these conditions encompass a large spectrum of clinical symptoms spanning from the milder Bethlem myopathy (BM) [2] to the very severe Ullrich congenital muscular dystrophy (UCMD) [2]. Further phenotypes include some intermediate forms of COL6-RM [3] and an additional, mostly contractural form described as myosclerosis myopathy (MM) [4]. A common feature of patients affected by Collagen VI defects is muscular weakness, which can progress slowly in BM or be congenital and rapidly progressive in UCMD, resulting in an early loss of ambulation. The most severe forms of COL6-RM also feature early and critical respiratory insufficiency. Proximal joint contractures and distal joint hyperlaxity are also common features in COL6-RM [2]. Specifically, joint contractures are defined as chronic reductions in the passive range of motion (ROM) of joints. Contractures result from structural alterations in the soft tissues surrounding joints, including muscles, ligaments, fascia, and tendons [5]. In the most severe cases, contractures completely prevent any movement of the affected joint. Notably, in dystrophic patients with minimal motor function, contracture of the thumb joint critically hinders the ability to independently control their wheelchair or to use a laptop, severely reducing their interaction with the outside world.

COL6-RM can be inherited in an autosomal dominant or an autosomal recessive manner. BM is usually inherited as dominant, although autosomal recessive inheritance has also been reported. UCMD and intermediate COL6-RM are typically caused by de novo autosomal dominant pathogenic variants of *COL6A1*, *COL6A2*, or *COL6A3* genes. Less commonly, UCMD and intermediate COL6-RM are inherited in an autosomal recessive manner. Parental somatic mosaicism and concomitant germline mosaicism are not uncommon in the autosomal dominant COL6-RM [2]. Mutations often occur in specific regions of the collagen VI protein, particularly within the globular domains or in the N-terminal part of the triple helical domain. While many different mutations are possible, some are more frequently observed. For example, a dominant glycine substitution (G293R) in *COL6A1* is one of the most common amino acid substitutions found in UCMD [6]. Additionally, some mutations are located near the C-terminal end of the triple-helical domain [2]. Besides missense variants, mutations can also lead to truncating variants, in-frame exon skips and deletions, and in-frame pseudoexon insertions [7]. In each case, in vivo and in silico studies have documented that mutations often impair the formation of collagen VI monomers, dimers and tetramers, with abnormal deposition and organization of collagen VI microfilaments in the ECM [8,9].

Collagen VI is typically reduced or absent in the basement membrane of muscle fibers of UCMD patients, while mild or undetectable changes of the protein pattern are usually associated with the BM phenotype [10]. Biochemical and morphological studies in cultured skin fibroblasts from UCMD patients have highlighted defects in collagen VI assembly and secretion, leading to an altered microfilament network [11,12]. In cultures of BM-derived skin fibroblasts, collagen VI microfilaments appear less affected, with minimal quantitative and structural changes [10]. Over the years, several studies have demonstrated that the pathological consequences of collagen VI alterations are not solely attributable to its reduced expression, but also to structural and functional changes of other ECM proteins that interact with collagen VI [1]. As a consequence of these alterations, the entire architecture of the ECM is impaired with detrimental effects on its physical-chemical properties, ultimately resulting in an altered transmission and adaptation to biomechanical and biochemical signals. Therefore, it is not surprising that the tissues most affected by collagen VI mutations are skeletal muscle and tissues most exposed to mechanical stimulation, including skin and tendons. Since the discovery of collagen VI involvement in this group of disorders, over ten years of research on tendon tissues and cultured cells from affected patients have yielded diverse, sometimes unexpected insights into the contribution of collagen VI defects in tendon dysfunction.

Morphological and biochemical evidence in COL6-RM patients and collagen VI-deficient animal models have documented cellular and ECM defects associated with decreased tensile strength and stiffness, suggesting tendon dysfunction. Studies performed on tendon-derived primary cultures from COL6-RM patients and pathological animal models strongly suggest that tendon alterations result from primary defects, rather than merely a consequence of the reduced muscular activity. Nonetheless, the results of such studies are somehow fragmented and poorly known. Over the years, in fact published reviews and original articles on collagen VI from the leading groups in the field [1,2,3,13,14,15,16] have consistently described collagen VI involvement in muscle, the tissue long considered the primary site of impact, but have largely overlooked its role in tendon. By integrating isolated findings and emphasizing tendon-specific alterations related to collagen VI deficiency, this review seeks to address knowledge gaps and promote progress in both research and clinical management of tendon pathology associated with collagen VI defects. The ultimate aim is to offer researchers, clinicians and patients up-to-date insights to facilitate the development of innovative experimental, diagnostic, and therapeutic approaches.

The review is structured into three main chapters. The first one, “Collagen VI in Normal Tendon”, discusses collagen VI assembly, structure, localization, and interactors in normal tendons. The second chapter, “Pathological Aspects”, examines pathological mechanisms in tendons and tendon cells from both COL6-RM patients and *Col6*-deficient animal models, focusing on morphological, biochemical, and biophysical defects, particularly regarding mechanical strain, with an emphasis on pathways downstream of focal adhesion and the primary cilium. The third chapter, “Collagen VI in Tendon Homeostasis”, explores the interplay between collagen VI and TGF-β1, a crucial regulator of tendon regeneration, healing, and formation, and between collagen VI and CMG2, a newly identified collagen VI receptor. The review concludes by speculating on how collagen VI dysfunction may contribute to tendon pathology, linking already reported and putative intracellular defects to tendon homeostasis.

## 2. Collagen VI in Normal Tendon

Tendons are structures mainly composed of dense connective tissue connecting muscles to bones. Facilitating the transmission of force generated by muscle contraction, tendons are essential for mobility [17]. Specialized regions known as the myotendinous junctions integrate and stabilize the insertion of the tendon to the muscle. At the same time, the osteotendinous junctions, also known as entheses, allow tendon attachment to the bone [17]. 

Tendons are primarily composed of parallel bundles of collagen fibers, which provide both strength and flexibility. The parallel organization of collagen fibrils allows tendons to withstand the tensile forces produced during muscular activity and effectively transmit these forces to the skeleton. An impaired organization of the bundles or of the single collagen fibers can affect the overall tendon function, increasing maladaptation to mechanical force and susceptibility to injuries [18].

Collagen fibers are formed through a continuous remodeling of proteins synthesized and secreted by tenocytes, which are specialized fibroblasts responsible for the production and organization of tendon ECM. Tenocytes localize among the collagen bundles, forming “columns” of cells arranged parallel to the fibers. This columnar arrangement facilitates cell contact with both the ECM and neighboring tenocytes, enabling communication and mechanosensation.

Tendons exhibit three different types of matrix, with unique composition and function [19].

The pericellular matrix (PCM) immediately surrounds tendon cells. It is rich in proteoglycans, including versican and aggrecan, glycoproteins such as perlecan, and collagen types VI and XI. PCM acts as a mechanosensory and regulatory niche that mediates cell-ECM communication. It stabilizes the environment around tenocytes, allowing cells to sense mechanical cues and regulate tissue homeostasis. Many PCM components interact with collagen VI to maintain matrix integrity and facilitate signaling pathways that influence tendon cell behavior and ECM remodeling [20].

The fascicular matrix (FM) is mainly composed of type I collagen fibrils, along with small leucine-rich proteoglycans (SLRPs) and other non-collagenous proteins that regulate collagen fibrillogenesis and organization. It provides the main tensile strength of the tendon due to highly aligned collagen fibrils. It maintains structural integrity and transmits mechanical loads along the length of the tendon [21].

The interfascicular matrix (IFM) is the softer matrix that separates and surrounds the fascicles within the tendon. It contains a higher proportion of glycoproteins, such as lubricin and elastin, collagen types III, V, VI and XII, fibrillins and small proteoglycans. This matrix facilitates sliding between fascicles and provides elasticity and recoil due to the presence of elastin and lubricin. It acts as a gliding system within tendons to accommodate complex mechanical loading and promotes resilience to fatigue and injury [21,22].

### 2.1. Collagen VI Assembly, Morphological Features, and Localization

**Figure 1:**
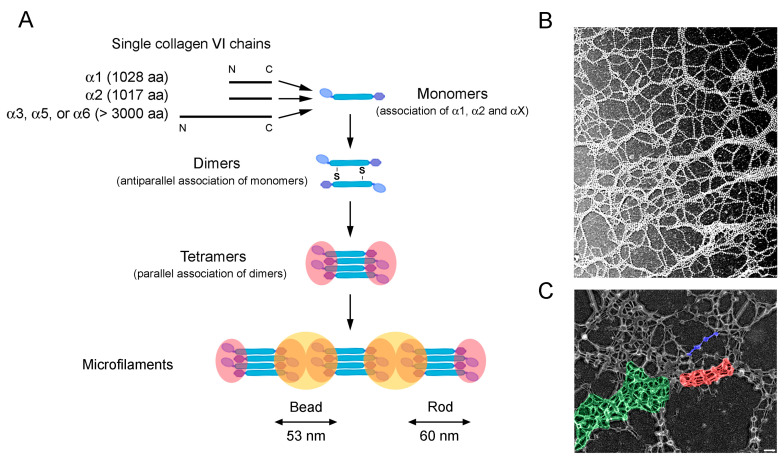
Synthesis and organization of collagen VI microfilaments. (**A**) Collagen VI polypeptide chains, including α1, α2, and the larger α3, α5 and α6 chains, are synthesized in the rough endoplasmic reticulum, where they undergo post-translational modifications, including hydroxylation and glycosylation. Three chains (where αX can be any of the larger α-chains) associate in a 1:1:1 ratio, forming triple-helical monomers. During subsequent maturation steps along the secretory pathway at the intracellular level, monomers further associate into antiparallel dimers stabilized by disulfide bonds (S). Dimers in turn align to form tetramers, which are finally secreted into the extracellular space. Outside the cell, tetramers self-assemble into characteristic beaded microfilaments that are deposited in the ECM. The canonical diameter of the beads, here highlighted by yellow circles, is 53 nm, while the rod region (in pink) is 60 nm [27]. (**B**) Transmission electron microscopy visualization of rotary shadowed replicas of cultured cell monolayer from human control donors immunolabeled with anti-collagen VI and 5 nm-colloidal gold conjugated secondary antibody, showing a branched network of collagen VI microfilaments in the ECM. This figure has already been published by Benati et al. [28]. (**C**) Transmission electron microscope visualization of rotary shadowed replicas of cultured tendon cell monolayer from human control donors, showing different structural organization of secreted collagen VI tetramers. By interacting through their globular domains, tetramers may assemble end-to-end, forming microfilaments (blue), or arrange radially, forming web-like (hexagonal) structures (green). In turn, parallel association of microfilaments forms broad banded fibrils (red). Scale bar 100 nm.

Collagen VI accounts for approximately 0.33% of tendon wet weight [23]. It localizes among collagen fibrils and appears particularly enriched in the PCM of tenocytes [24,25], the primary site of collagen fibril localization. Tenocytes, the main tendon cellular components, are responsible for the expression, secretion and assembly of the tendon ECM, including collagen VI. The most represented form of collagen VI is composed of α1, α2, and α3 chains, which associate in a 1:1:1 ratio intracellularly to form triple-helical monomers [1]. Due to sequence homology, the α5 and α6 chains may replace the α3 chain, forming α1α2α5 and α1α2α6 monomers, which, however, are less expressed and display a more restricted tissue distribution. While other mammals have a further α4 chain coded by a separate gene, in humans and great apes this chain is not expressed, as the corresponding CO008. L6A4 gene was part of a large chromosomal inversion during evolution, becoming a non-processed pseudogene [26]. Monomers then interact together in an antiparallel manner, forming disulfide-bonded dimers, which in turn form tetramers, stabilized by disulfide bonds. The central part of these tetramers contains the triple-helical domains and has a rod-like shape, flanked by two bead-like regions formed by the N- and C-terminal globular domains of the different α chain [1]. Finally, the tetramers are secreted into the extracellular space, where they associate end-to-end through non-covalent interactions, forming beaded microfilaments, which are a distinctive feature of collagen VI (Figure 1). The extracellular deposition of collagen VI is also supported by cell surface proteins, including α_1_β_1_, α_2_β_1_, and α_10_β_1_ integrins and NG2/CSPG4 proteoglycan, which help in promoting the organization and assembly of collagen VI microfilaments into larger networks in close vicinity to the cell surface [1,27].

Besides the canonical branched network of beaded microfilaments [27], collagen VI tetramers may be assembled into other forms, including broad banded structures [29] and hexagonal networks [30], as observed in vitro when incubated with biglycan [30] or in tissue cultures (Engvall 1986). These data indicate that collagen VI microfilaments may change their three-dimensional arrangement depending on specific functional roles. It is conceivable that these different arrangements are driven by the interactions with specific binding partners. The supramolecular organization of collagen VI in tendon ECM is shown in Figure 1B,C.

Early ultrastructural studies on collagen bundles isolated from mature tendons described collagen VI beaded microfilaments running parallel to collagen I fibrils [12]. In tendon fibroblast cultures, web-like structures composed by collagen VI microfilaments are closely associated with the cell surface, forming a pericellular scaffold [31]. In vitro studies detected anchoring bundles of parallel-aligned collagen VI microfilaments at the trailing edge of migrating cells [20].

**Figure 2:**
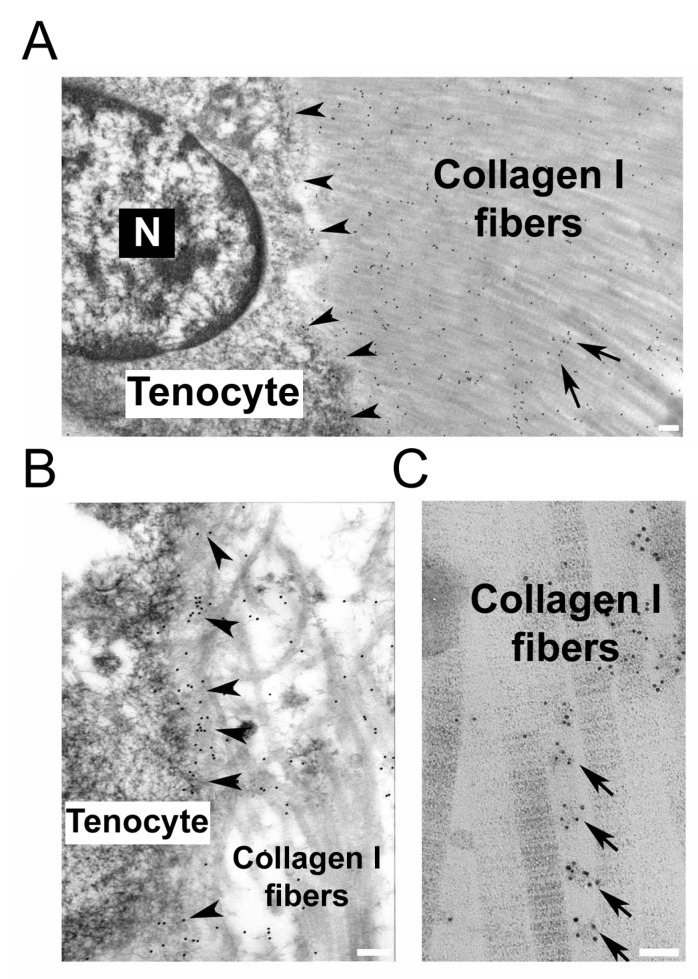
Collagen VI localization in tendon. (**A**) Immunoelectron microscopy of collagen VI α3 chain and 10 nm colloidal gold antibody in a longitudinal section of human tendon. Note that colloidal gold particles are detected both in the pericellular matrix (arrowheads), closely associated with the tenocyte surface, and among collagen I fibers of the fascicular matrix (arrows). N, nucleus. (**B**) Magnified detail of the pericellular matrix (arrowheads) of a tenocyte, highlighting the high concentration of the colloidal particles identifying collagen VI closely associated with the cell membrane. (**C**) Magnified detail of the fascicular matrix of a normal tendon showing the periodic distribution of collagen VI (a3 chain) between the collagen I fibers (arrows). Scale bar, 1 µm (top and bottom left panel) and 100 nm (bottom right panel).

Figure 2 shows the localization of collagen VI in control tendons.

**Figure 3:**
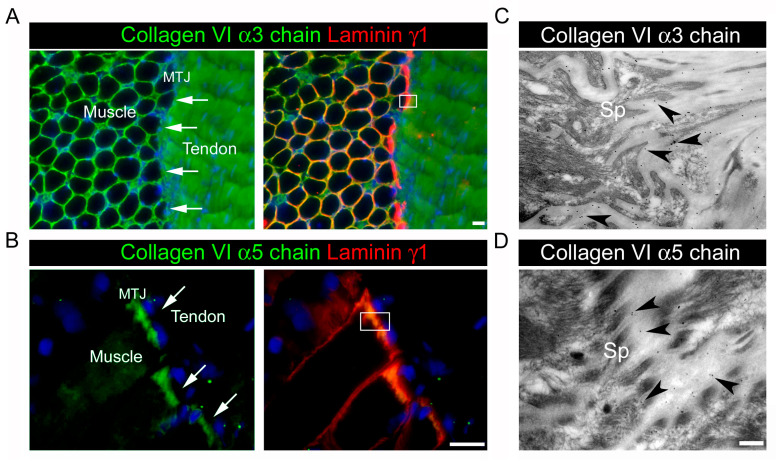
Collagen VI localization at the myotendinous junctions. (**A**) Immunofluorescence microscopy of a transversal section of a biopsy of a normal muscle showing the myotendinous junctions (MTJs), the interface between muscle and tendon. MTJs are indicated by the arrows. Collagen VI was stained by an antibody against collagen VI α3 chain (green), while the basal lamina of muscle fibers and of the MTJs by laminin γ1 (in red). Nuclei were stained with DAPI (blue). (**B**) Immunofluorescence microscopy of a longitudinal section of a biopsy of a normal muscle showing the myotendinous junction (MTJ, arrows), in which collagen VI α5 (in green) co-localizes with laminin γ1 (red). Nuclei were stained by DAPI (blue). (**C**,**D**) Immunoelectron microscopy of collagen VI α3 (**C**) and α5 (**D**) chains and 10 nm colloidal gold antibody (black dots, arrowheads) at the insertion of a normal tendon with muscle. Both images are representative of the areas outlined by the white box in (**A**) and (**B**) respectively. Arrowheads point to colloidal gold particles associated with the sarcolemma projections (Sp) with the typical finger-like aspect at the MTJ. Scale bar, 100 nm.

In mature tendons, two forms of collagen VI microfilaments are produced. The α1α2α3, the most abundant, is widespread in the tendon matrix, among the collagen I fibers of the FM, being particularly enriched in the PCM, where it forms a microfibrillar scaffold around the cells. The α1α2α5 is selectively expressed at the myotendinous junctions, and specifically in the ECM associated with the finger-like projections of the myofiber sarcolemma (Figure 3).

### 2.2. Collagen VI Interactors

In vitro and in vivo studies have shown that collagen VI interacts with a range of proteins located in the PCM, FM and IFM, as well as with several cell surface receptors. These interactions can occur directly or be mediated by extracellular binding partners. A list of collagen VI tendon interactors is provided below.

1. Type I collagen. In tendons, collagen I is the major fibrillar collagen that functionally interacts with collagen VI, contributing to the organization and mechanical properties of the ECM [32]. Tendon cells display a partial co-distribution between collagen I and VI [8,20]; however, the precise molecular mechanisms by which collagen VI modulates collagen I fibril assembly are not known. These interactions are likely mediated by changes in the expression of molecules that regulate collagen fibrillogenesis [33].
2. Type XII collagen. Collagen XII belongs to the family of fibril-associated collagens with interrupted triple helices (FACIT) and consists of a homotrimer of α1(XII) chains at the C-terminus. It is largely expressed in tendons, where, by interacting with collagen I through the collagenous domain, it regulates fibrillogenesis. There is evidence for a physical interaction between collagen XII and collagen VI [34], however, a direct or ternary structural interaction between these two collagens has not yet been reported [35,36].
3. Fibronectin is an adhesive glycoprotein that connects cells to the ECM and facilitates the polymerization of collagen fibrils. Fibronectin also contains RGD sequences recognized by integrin receptors such as α5β1 [11]. Collagen VI has been shown to co-localize with fibronectin in the basement membrane, regulate epithelial cell interactions with fibronectin [37], and facilitate the three-dimensional organization of fibronectin within the ECM [11]. Direct binding of collagen VI to fibronectin was reported in some studies using recombinant proteins [38,39], and fibroblasts lacking collagen VI display altered fibronectin deposition [11]. In normal tendon cultures, a fine network of intertwined fibronectin fibrils has been detected, which co-localize with collagen VI microfilaments [20].
4. Collagen IV. Collagen VI has been shown to physically interact with collagen IV, a major component of basement membranes [1]. Through its direct interaction with collagen IV, collagen VI is potentially involved in maintaining the stability of tendon endothelial cells and blood vessels. In addition, collagen VI binding to collagen IV plays a pivotal role at the basement membrane of myotendinous junctions, stabilizing the muscle-tendon interface [40], the primary site of force transmission. Furthermore, by binding collagen IV, collagen VI is anchored to other proteins of the basal membrane, including laminins and entactins [41], contributing to the structural scaffold of the ECM.
5. Vitronectin modulates matrix elasticity and degradation. Like other collagens, collagen VI binds to vitronectin, affecting ECM stability and remodeling processes [42].
6. Glycosaminoglycans (GAGs) and proteoglycans contribute to the three-dimensional organization of the ECM, as well as to its biomechanical properties and hydration. Some studies reported the association of keratan sulfate and dermatan sulfate GAGs with collagen VI in the cornea [43]. Hyaluronan and heparin are two other GAGs that associate with collagen VI and influence matrix architecture and dynamics. On the other hand, biglycan and decorin, two small leucine-rich proteoglycans, have a major role in the maintenance of mature tendon mechanics [44], and interact with collagen VI [32,45]. Collagen VI and biglycan co-localize within nascent tendon matrix, indicating possible synergy in tissue function [32]. Finally, perlecan, a large heparan sulfate proteoglycan highly expressed in tendons and involved in fibrillogenesis, physically interacts with collagen VI [46,47].
7. Elastic fibers are key contributors to tissue elasticity, which indirectly interact with collagen VI, therefore supporting matrix organization and contributing to the mechanical resilience of tissues. Among the components of the elastic fibers, microfibril-associated glycoprotein 1 (MAGP1) has been shown to interact with collagen VI through direct binding to the triple-helical domain [1].
8. Integrins are the main class of cell surface proteins acting as adhesion receptors for different ECM components, providing both biomechanical and biochemical signals for cells. Integrins transduce signals bidirectionally, as they can transmit information from the ECM to within the cell and from the cell to the ECM. Integrins are dimers composed of an α and a β subunit, which differ between the various members of the integrin family, thus ensuring ligand binding specificity. Multiple evidence suggest that the triple helical region of collagen VI binds several integrins, such as α_1_β_1_, α_2_β_1_, α_10_β_1_, α_11_β_1_ and α_v_β_3_ [48,49,50], but not as an exclusive ligand. Integrins were also reported to contribute to the proper assembly of collagen VI into mature microfilaments in the extracellular space [27]. In tendon cells, integrins, especially those belonging to the β_1_ subfamily, play a crucial role in mechanotransduction and ECM interactions [51]. Moreover, collagen-binding integrins α_1_, α_2_, and α_11_ are upregulated in tendon stem/progenitor cells in response to mechanical stimulation, playing roles in sensing mechanical forces and regulating matrix remodeling [52]. Finally, α_5_β_1_ and α_v_β_3_ integrins increase their expression in tendon cells upon growth factor stimulation induced by regenerative stimuli [53].
9. Chondroitin sulfate proteoglycan 4 (CSPG4), also known as neural/glia antigen 2 (NG2), is a transmembrane protein acting as a collagen VI receptor [31]. Interaction of CSPG4/NG2 with collagen VI facilitates the structural organization of the ECM and the cell-ECM adhesion [25], as well as the activation of key signaling pathways [54]. Previously, we demonstrated that the collagen VI-NG2 binding is crucial for the proper migration of tendon cells. By anchoring the cell to the substrate through its interaction with NG2, collagen VI contributes to cell polarization and efficient movement [31].
10. Anthrax receptor 2/capillary morphogenesis gene 2 (ANTXR2/CMG2) is a transmembrane receptor binding to the triple helical domain of collagen VI. This interaction leads to endocytosis and lysosomal degradation of collagen VI, playing a key role in collagen VI turnover and homeostasis [55]. In contrast, ANTXR1/TEM8 plays an uncertain role in collagen VI binding. Although ANTXR1/TEM8 was originally reported to bind the C-terminal C5 domain of the α3 chain, which is typically cleaved off during collagen VI maturation, recent studies have found no evidence of binding between fully assembled collagen VI or the cleaved C5 fragment (also known as endotrophin), neither in tendon cells nor in other cellular models [56].

**Figure 4:**
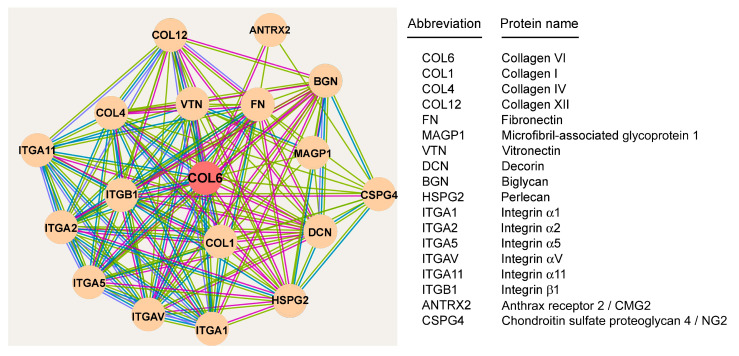
Protein network formed by collagen VI in tendon cells. The diagram shows the collagen VI interaction network within the tendon ECM and on tenocytes’ surface. Edges indicate both functional and physical protein associations. Light blue and fuchsia edges denote interactions sourced respectively from curated databases and experimental evidence. Lime-colored edges indicate interactions inferred from text-mining analyses. The interaction confidence score threshold was set at 0.2. The network is centered on the collagen VI α1 chain (in red at the center). For clarity, collagen VI α2, α3, and α5 chains are not shown. The network was generated using STRING version 12.0.

The interactome of collagen VI in tendon cells is summarized in Figure 4.

## 3. Pathological Aspects

### 3.1. COL6-Related Tendon ECM Dysfunction

Studies based on tendons and tendon cells from COL6-RM patients and from collagen VI-deficient animal models showed disrupted collagen I fibrillogenesis, altered tenocytes morphology, and decreased mechanical strength of tendons. The presence of mutated chains or the complete absence of collagen VI leads to an impaired maintenance of tendon collagen I fibers, resulting in reduced tissue integrity. These findings have stimulated considerable interest and debate regarding collagen VI’s role in the synthesis and structural organization of the ECM, as well as in the regulation of tendon homeostasis.

#### 3.1.1. Evidence from Animal Models

Currently, genetically engineered mice remain the most commonly used in vivo model for investigating disease pathogenesis and molecular mechanisms in COL6-RM. In mice, collagen VI deficiency leads to significant alterations in tendon structure and function. Over the years, three different mouse models with absent or defective collagen VI expression have been developed. These models were generated by different research groups through the deletion or mutation of one of the three most important chains [33,57,58]. From a clinical point of view, all these models display phenotypic features resembling BM and UCMD.

In 2010, using the *Col6a1* null mouse model developed in Paolo Bonaldo’s laboratory, David Birk’s team demonstrated dysfunctional tendon collagen fibrillogenesis [12]. While in wild-type mice, tendon fibroblasts were well-organized with defined microdomains containing collagen I fibers, in *Col6a1* null mice these microdomains were disrupted, with less organized fibers and altered fibroblasts morphology showing numerous cellular processes with thin branches [12]. Tendons from *Col6a1* null mice also showed smaller collagen I fibril diameter, abnormal fibril structure, particularly in pericellular regions, and increased fibril density. Biomechanical testing revealed decreased tensile strength and stiffness, indicating that collagen VI contributes to the maintenance of the mechanical properties of tendons. Altered expression of matrix metalloproteinases was also detected, pointing at abnormal remodeling of ECM and cell-ECM interactions [12]. Comparable alterations were further reported by other independent studies, in which it was shown that tendons from *Col6a1* null mice exhibit ECM dysregulation, microarchitecture disruptions, and functional defects [59]. Similarly, tendons from *Col6a2* null mice were found to have decreased cross-sectional area, as well as stiffness and resistance to mechanical load [58]. Comparable findings were obtained in a *Col6a3* mutant mouse model expressing a very low level of a non-functional α3 chain, with abnormal collagen I fibrils in tendons, but not in cornea, indicating a distinct tissue-specific effect of collagen VI on collagen I fibrillogenesis [33].

In zebrafish, tendon populations are identified in the craniofacial and pectoral fin, and in myoseptal regions [60]. Besides significant muscular alterations, *col6a1* null zebrafish have altered myosepta structures which contribute to impaired muscle organization and compromise the transmission of mechanical force [61]. *Col6a1* morphant fish, produced by morpholino antisense oligonucleotide-mediated knockdown, also present multiple abnormalities at the myotendinous junctions, including a less dense collagenous matrix and swollen/dilated endoplasmic reticulum [62]. Similarly, *Col6a2* morphant fish, besides muscle alterations, also display misshapen and abnormally ramified vertical myosepta [63]. Altogether, these findings demonstrate that collagen VI deficiency affects the organization of the tendon fibroblasts, the pericellular collagen fibril-forming microdomains defined by the tenocyte surface, and ultimately the mechanical properties of tendons.

#### 3.1.2. Additional Animal Models

Collagen VI deficiency has also been reported in dogs, in particular in Landseer, due to a homozygous mutation in the *COL6A1* gene (mutation p.Glu97*) [64], and in some Labrador retriever families, caused by a recessive mutation in the *COL6A3* gene (mutation p.R1576*) [65]. These dogs have congenital myopathy with joint contractures and hyperlaxity of distal joints, making them a valuable large animal model for COL6-RM. Currently, there is no evidence of functional alterations in tendons or tendon cells in these models, as the available information is limited to the study of muscle morphology and activity.

#### 3.1.3. Evidence from COL6-RM Patients

Since the diagnosis of the first UCMD and BM patients, which occurred in 1930 and 1975 respectively, and the genetic identification of the causative mutations of BM in 1996 [66] and of UCMD in 2001 [67], significant progress has been made in elucidating the pathogenic mechanisms underlying the onset and progression of clinical symptoms in this group of disorders. In particular, in vitro studies with patient-derived muscle cells allowed us to confirm the pathomolecular pathways previously identified in the *Col6a1* mouse model, as well as to explore potential targets for therapy. This kind of approach was fundamental for the design of pilot clinical trials based on the use of cyclosporin A [68] to reverse the mitochondrial dysfunction found in muscle cell cultures from *Col6a1* null mice and UCMD patients [69], or on the application of a low-protein nutritional approach [70], to counteract the autophagic defects found in myofibers of *Col6a1* null mice [70] and BM/UCMD patients [71]. Similarly, the study of pathogenic tendon cells cultured under experimental conditions that mimic key tendon functions has not only contributed to increasing knowledge about the role of collagen VI in various cellular processes, but also provided insights into pathways for ameliorating the pathological phenotype.

One of the first findings detected in UCMD-derived tendon cultures was defective collagen VI organization. In these cultures, collagen VI showed abnormal intracellular retention [25]. Moreover, instead of the typical microfilamentous network, UCMD tendon cultures showed extracellular aggregates [8,25]. Interestingly, collagen VI abnormalities were also linked to defects in the organization of collagen I, collagen XII, and fibronectin [8,25]. Similar to tendons of *Col6a1* and *Col6a2* null mice [12,58], ultrastructural analysis of UCMD tendon tissue confirmed irregular collagen fibril profiles and reduced fibril diameter [25]. Additionally, the association of collagen VI with collagen I fibril bundles was markedly impaired, when compared to the intense labeling seen in normal tendon ECM. Immunoelectron microscopy studies also revealed a significant reduction of collagen I and fibronectin in the PCM of UCMD tendon fibroblasts [25]. Increased MMP-2 activity was observed in pathological cultured tendon cells, indicating active remodeling and degradation of ECM components [25]. ECM defects, including aggregates and disrupted collagen VI organization, were also found in BM tendon fibroblasts [31].

As stated above, ECM composition directly affects its own stiffness and viscosity, which in turn influence the functions of ECM itself. At the same time, the presence of aggregates due to impaired fibrillogenesis and possibly to altered internalization and degradation of mutated microfilaments (see below) can negatively impact the activation of signaling cascades related to mechanical strain, as well as cell adhesion and migration in response to injury or regenerative stimuli [72].

### 3.2. Collagen VI in Cell Migration

Cell migration is regulated by several mechanical parameters, such as surrounding confinements, stiffness and the cell rear contraction [73]. In this context, defects in collagen VI and the collagen VI-related network can lead to detrimental consequences in the proper positioning and migration of cells. Tendon cells derived from UCMD and BM patients exhibit impaired polarization and migration when induced to migrate in a wound-healing assay [20]. Specifically, collagen VI defects were found to cause patients’ cells to close the wound in a random trajectory compared to unaffected control cells [20]. A mechanistic explanation for this defect was found in the impaired binding between collagen VI and its receptor NG2, which was instead evident at the trailing edge of migrating cells in control donor samples [20]. In response to injury, tendon fibroblasts are activated, migrate to the wound, and contribute to tissue repair by producing and organizing the ECM. Therefore, it is conceivable that by anchoring the cell rear to the substrate, collagen VI contributes to the stability of cell direction, suggesting a critical role for collagen VI in the machinery activated for tendon repair [20].

### 3.3. Collagen VI Role in Mechanotransduction

Mechanotransduction is the process by which cells convert extracellular mechanical stimuli into intracellular biochemical signals, ultimately activating genes that drive cellular adaptation to stress. This mechanism is exquisitely fine-tuned, such that even subtle alterations in the functions of each mechanotransducer can have detrimental consequences. At the molecular level, mechanical strain propagates through the active remodeling of ECM components, which is specifically detected by cellular mechanosensors, including focal adhesions and primary cilia. In the following paragraphs, we describe significant new aspects of the molecular response to mechanical stimulation in the context of a collagen VI-deficient ECM.

#### 3.3.1. Focal Adhesion Mediated Signals

In a recent work, we investigated how primary tendon cells from UCMD patients respond to mechanical strain. This stimulation was applied using a tension system that closely replicates the sustained and uniaxial strain that tendons normally experience [8]. Our findings revealed that UCMD-derived tenocytes exhibit an altered mechanoresponse [8]. Following strain application, UCMD cells displayed a reduced number and altered distribution of focal adhesions. UCMD cells also showed slower activation of signaling pathways driven by FAK, Akt, ERK1/2, and p38MAPK. Intriguingly, patients’ cells exhibited reduced YAP activation, which was consistently paralleled by decreased expression of YAP-responsive genes [8].

**Figure 5:**
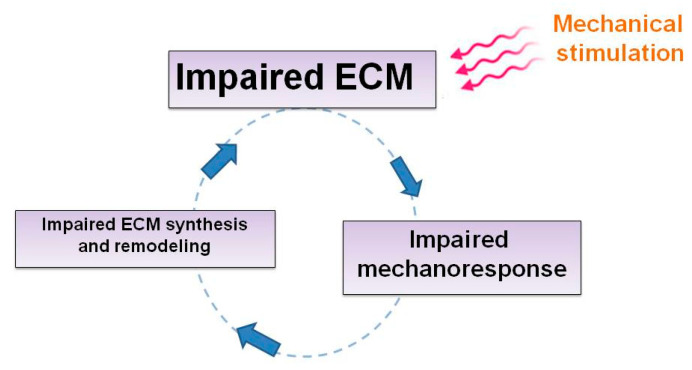
Schematic diagram depicting how a hypothetical feedback loop may self-sustain the impaired ECM organization in collagen VI-deficient tendons. A positive feedback loop, highlighted by both blue arrows and dashed lines between the impaired ECM (due to collagen VI defects) and the impaired mechanoresponse displayed by COL6-RM-derived tendon cells [8] might exacerbate the biochemical and biomechanical properties of the ECM, upon the arrival of mechanical stimuli (pink curly arrows) leading to a self-sustained deterioration of the ECM.

Collectively, these findings strongly support the concept that the ECM alterations elicited by collagen VI deficiency lead to abnormal mechanical signal transmission in tendon cells of UCMD patients. Interestingly, among the known YAP targets are genes coding for proteins involved in ECM regulation and remodeling, such as connective tissue growth factor 2 (CTGF2), CYR61 and MMPs. These genes play key roles in cell proliferation, migration, and ECM interactions under various physiological and pathological conditions [74,75]. Further research is needed to confirm the presence of a positive feedback loop, whereby mechanical strain on the already compromised ECM in tendons of COL6-RM patients induces progressive deterioration via disrupted mechanotransduction (Figure 5).

Confirming the crucial role of collagen VI in the whole ECM, our results further highlighted that in response to mechanical stimulation, UCMD tendon cells featured an altered dynamics of the actin skeleton network [8]. This aspect is particularly relevant in tendon cells, where actin stress fibers regulate cell homeostasis through the modulation of several key pathways [76].

#### 3.3.2. Primary Cilium Mediated Signals

Primary cilium (PC) is a mechanosensory organelle that protrudes from the cell surface into the ECM [77,78]. Through the presence of several membrane receptors, the PC can capture molecular changes in the ECM, including changes in the levels and post-translational modifications of its components, which generally occur upon strain [78]. Several studies have demonstrated that the PC is involved in cell proliferation, differentiation, migration, mitochondrial function and autophagy, as well as in ECM remodeling and mechanotransduction [79]. The central role of PC is demonstrated by ciliopathies, a group of genetic disorders caused by mutations in genes encoding for ciliary proteins. Ciliopathies exhibit defects affecting multiple organs, including the skeletal system [80,81].

Our work revealed that in tendon cells from UCMD and BM patients, the PC is longer (~30%) than in unaffected control samples, suggesting possible alterations in molecular pathways related to PC [8]. Of note, UCMD tendon cells fail to rescue cilia length after strain application, paralleled by impaired activation of Hedgehog (Hh) signaling, a pathway specific mainly for PC [8]. Hh signaling is known to be activated by secreted ligands, namely Shh, Ihh, and Dhh, and is essential during organogenesis and for maintaining cell phenotypes in differentiated tissues [82]. In tendons, it was demonstrated that Hh signaling is crucial during embryonic development for specifying the differentiation and promoting the maturation of tendon entheses [83]. Pharmacological or genetic modulation of Hh signaling in mice leads to defects in mechanical strength and impaired tendon enthesis healing [84].

In their whole, this evidence well describes the need for balanced regulation of PC and the Hh pathway for healthy tendon function and repair, and identifies PC and its related pathway as novel bona-fide targetable molecular mechanisms.

## 4. Collagen VI in Tendon Homeostasis

### 4.1. Interplay Between Collagen VI and TFG-β1 Signaling

TGF-β1 is essential for tendon formation, healing, and ECM remodeling [85]. TGF-β1 plays a critical role in tendon cell differentiation during the early stages of tendon development or upon regeneration, inducing the expression of two tendon-specific genes, *SCX* and *TNMD*, which encode scleraxis and tenomodulin, respectively [86,87]. TGF-β1-related activation of Smad3 is also crucial for ECM organization and tenocyte activity in response to mechanical stimulation [88]. It has been reported that, upon strain application, TGF-β1 levels increase and are related to the upregulation of collagen I as well as of MMP-2 and MMP-9, which drive the proteolysis of inactive pro-collagen I and the release of mature and active collagen I [89]. In this context, TGF-β1 plays a crucial role in ECM remodeling.

It is interesting to note that TGF-β1 promotes the trans-differentiation of fibroblasts to myofibroblasts, which is one of the most important features of scar fibrosis formation [90]. Myofibroblasts are fibroblasts with characteristics of smooth muscle cells, expressing α-smooth muscle actin and developing a contractile apparatus. Activated myofibroblasts generate traction forces required for wound closure and ECM remodeling [91]. However, hyper-activation of myofibroblasts induces tendon contractures [91] (see also below).

TGF-β1 is initially produced as an inactive precursor, which is processed into a dimer and secreted into the ECM in a latent form, where it remains associated with other proteins. Therefore, the ECM, by facilitating interactions between TGF-β1 and some matrix components, such as fibrillin-1 and fibronectin, serves as a local reservoir of latent TGF-β1 [92]. A recent study has shown that in wild-type mice, collagen VI forms a complex with active TGF-β1. However, in collagen VI-deficient mice there is a marked alteration in TGF-β1 binding partners, which is associated with an altered TGF-β1 signaling response. These findings strongly suggest that collagen VI plays a key role in modulating TGF-β1 bioavailability in response to specific stimuli [93]. Previously, we have demonstrated that TGF-β1 treatment of normal tendon cells dramatically reduces the expression of the α5 chain of collagen VI, while upregulating the expression and extracellular release of the α6 chain [94]. These findings indicate that collagen VI α5 and α6 chains are differentially involved in tendon matrix homeostasis and further emphasize the importance of TGF-β1 signaling in regulating ECM remodeling [94]. Interestingly, at the steady state, an upregulation of the TGF-β pathway has been observed in muscles from COL6-RM patients and from *Col6a2* null mice [93,95].

Overall, these observations suggest that in tendons and in cultured tendon cells, TGF-β1 signaling might be impaired, negatively affecting tissue differentiation, regeneration, and healing responses, which may also represent a risk factor of joint contractures (see below).

### 4.2. Collagen VI Turnover by CMG2

Recently, it has been demonstrated that the cell surface receptor ANTXR2/CMG2 plays a crucial role in the homeostasis of collagen VI by regulating its extracellular abundance [55]. Mutations of the *ANTXR2* gene cause hyaline fibromatosis syndrome (HFS), a severe genetic disorder characterized by the abnormal accumulation of hyaline material in the skin, *mucosae*, and various internal organs, in the form of subcutaneous nodules and plaques [96]. Loss of function of CMG2 promotes the accumulation of collagen VI in the skin of HFS patients as well as in *Cmg2* null mouse models [55]. Recent studies have demonstrated that collagen VI binding to CMG2 leads to an increase in CMG2 phosphorylation/activity, followed by ligand-induced endocytosis of the receptor, which targets collagen VI to lysosomal degradation [97].

Altogether, the evidence reported so far demonstrates the centrality of the role of collagen VI in ensuring the proper functioning of tendons.

## 5. Insights into the Potential Contribution of Pathogenic Mechanisms to Tendon Dysfunction

Joint contractures are a distinctive trait of muscular dystrophies linked to collagen VI defects [98]. They are caused by shortening and hardening of the tissues surrounding the joints, including muscles, ligaments, fascia, and tendons [5]. 

By developing an animal model deficient of *Smad4* in the scleraxis cell lineage (i.e., specific for tendons), recent studies have evidenced that joint contractures are associated with alteration of the activity of the Smad4-dependent signaling cascade in tendon cells [99,100]. Intriguingly, the authors also reported that Smad4 activity is required for a proper tendon fibrillogenesis [99]. In agreement with the altered TGF-β1 levels in muscles of collagen VI-deficient mice [93] and COL6-RM patients [95], data from our team suggest that primary tendon-derived cultures from COL6-RM exhibit increased TGF-β1 expression compared to controls (V. Cenni and P. Sabatelli, unpublished). Considering that Smad4 is a transcription factor transducing TGF-β1-related signaling, our unreported findings suggest that COL6-RM patients-derived tendon cells present an abnormal activity of Smad4 that may contribute to the onset of joint contractures in these disorders.

As described above, altered collagen I fibrillogenesis is also a striking hallmark of COL6-RM tendons and cultured tendon cells. By impairing the physical properties of the ECM, improper fibrillogenesis and/or matrix remodeling results in the alteration of the mechanoresponse of tendons [101], as well as an impairment of tendon function, possibly contributing to the onset of joint contractures. Of note, an abnormal change in ECM stiffness has been observed in matrices from COL6-RM fibroblasts [102] and in *Col6a1* null mouse tendons [12].

The altered stiffness of the ECM of COL6-RM tendons may stem from a range of disrupted mechanisms, including the internalization and/or degradation of mutated collagen VI. For example, changes in the steric conformation of mutated collagen VI in COL6-RM may lead to the inability of CMG2 to correctly recognize collagen VI [55], leading to an impaired internalization of collagen VI, and to its harmful accumulation in the extracellular space.

Autophagy plays a crucial role in maintaining ECM homeostasis in tendons [103], and an abnormal regulation of the autophagic flux shifts the size distribution of collagen I fibrils, resulting in a reduction of mechanical strength under tensile force [103]. It is very well known that collagen VI has a pro-autophagic role [104,105] and that muscles from *Col6a1* null mice and from COL6-RM patients have impaired autophagy [71,105]. Therefore, an impaired autophagic activity cannot be excluded in tendons. Impaired autophagic activity may have severe consequences in the regulation of ECM quality and function. Besides the established role of autophagy as a regulator of tendon homeostasis [103] autophagic defects may exacerbate the effects of TGF-β1 in ECM remodeling [106], and contribute to an improper degradation of mutated collagen VI via CMG2 [55,97].

Concerning other unexplored mechanisms that could alter the turnover of ECM components and therefore ECM stiffness, it is important to consider that some forms of ciliopathies, including Meckel-Gruber syndrome (MKS) and skeletal ciliopathies, including Jeune chondrodysplasia and short rib-polydactyl syndromes, exhibit joint contractures as part of their phenotypic spectrum [107]. These conditions typically involve defects in ciliary function associated with impaired ciliary-dependent mechanotransduction and changes in ECM composition that result in defective skeletal development, with restricted joint mobility and deformities [107]. In this context, the functional alteration of the primary cilium observed in tenocyte cultures from COL6-RM patients [8] might contribute to the development of contractures.

In tendons, the enthesis, the interface where tendons, ligaments, or joint capsules attach to bone, plays a crucial role in joint stability and mobility by effectively transferring muscle forces to the skeleton. This function is supported by the fibrous architecture of the enthesis, which is essential for resisting injury and maintaining precise joint movement [108]. Molecular changes or structural alterations at the enthesis can compromise this mechanical interface, leading to tendon dysfunction and joint stiffness, which may contribute to the development of joint contractures. The Hh signaling pathway is necessary for tendon enthesis development [83]. Specifically, finely tuned regulation of Hh signaling during development drives the formation of a mineral gradient across the tendon enthesis fibrocartilage [83]. The alteration of the primary cilium and Hh pathway observed in COL&-RM tendon cells [8] may interfere with enthesis activity and contribute to the pathogenesis of joint contractures. A similar defective mechanism might drive the formation of dysfunctional myotendinous junctions (MTJ), the transitional zones where tendons attach to muscles. In a recent imaging study performed at the muscular level, abnormalities were reported in the MTJ of triceps brachii, iliopsoas, adductor longus and gastrocnemius muscles. In the latter, MTJ alterations significantly correlated with ankle contractures [109]. 

Future studies, possibly exploiting 3D models, could prove crucial in validating these hypotheses. On the other hand, in order to provide a detailed structural assessment and thus new insights into tendon dysfunction in patients affected by COL6-RM, it would also be advisable to incorporate high-resolution imaging studies of tendons near contractures and hyperlaxities in future studies.

## 6. Therapeutic Perspectives

The clinical management of skeletal muscle symptoms in COL6-RM currently focuses on supportive care, as there is no definitive cure yet. Experimental antisense oligonucleotide-based therapies are being developed to correct specific mutations in COL6 genes, with the aim of effectively restoring a functional collagen VI protein in patients’ cells [110]. However, the processes through which these DNA- or RNA-modifying oligonucleotides are delivered to patients have not yet been optimized and are therefore the subject of ongoing studies. At the same time, studies and clinical trials are underway to target the downstream cellular and molecular mechanisms affected by collagen VI deficiency, in particular defective autophagy and mitochondrial dysfunction, with the aim of counteracting disease progression or improving muscle function.

In the context of joint contractures, the current clinical care in the early stages of the disease focuses on intensive physical therapy aimed at relieving contractures through exercises that strengthen muscle trophism and stretch tendons. Orthopedic surgeons can contribute by correcting the severe deformities of the spine and extremities, typical of COL6-RM patients, and by adopting less invasive approaches to promote rapid recovery and more effective post-surgical care [15]. However, these approaches have had only limited success.

Recent advances in understanding tendon cell pathology, ECM homeostasis regulation, and repair mechanisms have identified several promising therapeutic targets, including modulation of ciliary function and the Hh signaling pathway, the TGF-β signaling cascade, and the mechanotransduction pathways. Future studies focusing on patient-derived cells, animal models, and gene/transcript-editing technologies will be essential to translate these findings into effective clinical therapies.

## 7. Conclusions

**Figure 6:**
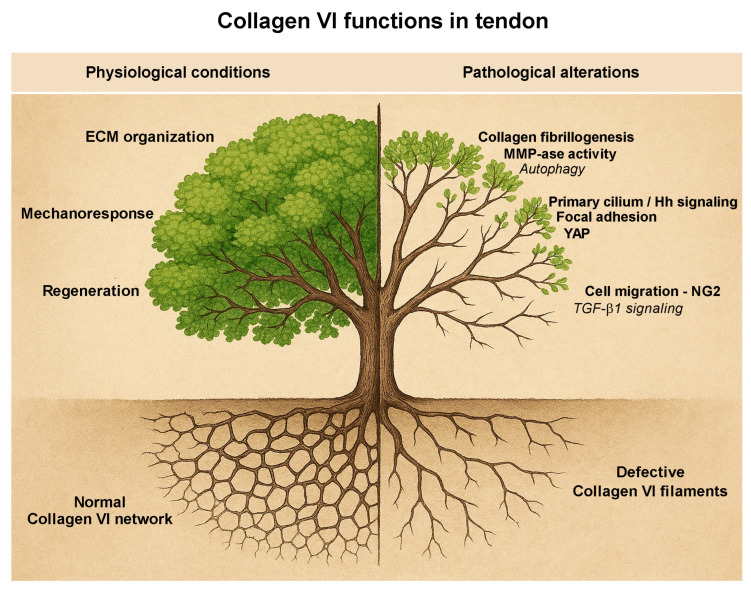
Metaphorical representation of collagen VI functions in tendons. A fully functional collagen VI network, depicted as a complex root system on the left side of the tree, supports healthy cells and tissue, represented by the richly green portion of the tree. Collagen VI guards the proper organization of the ECM, and mediates the appropriate response to both mechanical and regenerative stimuli. In contrast, mutated collagen VI, illustrated by fragmented roots on the right side, leads to defective fibrillogenesis and increased metalloprotease activity, disrupted signaling pathways downstream of the primary cilium and focal adhesions, and impaired cell polarization during migration. Collectively, these dysfunctions result in weakened cells and tissue, symbolized by the less lush portion of the tree on the right. Processes written in italics (i.e., Autophagy and TGF- β1 signaling) have to be intended as collagen VI-related defects that remain unconfirmed in tendons. Abbreviations: ECM: extracellular matrix; MMP-ase: Matrix metallo-proteinase; Hh: Hedgehog; NG2: Nerve-Glial antigen 2 proteoglycan. The image was created with the assistance of Microsoft Copilot (Office 365).

Tendons and tendon cells are crucial in the pathogenesis of COL6-RM. Recent advancements in cellular and molecular studies, especially those utilizing human pathological models, have enhanced our understanding of tendon dysfunction associated with this condition. This new perspective identifies the tendon as a primary contributor to musculoskeletal defects, contrasting with the earlier belief that it was merely a secondary effect. However, the limited number of studies specifically focusing on tendons, and on primary tendon cultures from COL6-RM patients or Col6-deficient animal models, along with only incidental observations in muscle-centered research, reveal a critical gap in the knowledge of these pathologies, underscoring the need that motivated this work to integrate these fragmented and seemingly disconnected data. Evidence from biochemical and morphological changes observed in COL6-RM tendon cultures -including impaired responses to mechanical stimuli and TGF-β1, defective cell polarization, disrupted focal adhesion distribution, altered primary cilium morphology, and dysfunctional downstream signaling pathways- reveals novel actionable targets for intervention, including, for example, modulation of mechanotransduction components which remain underexplored in patient-derived tendon models. Figure 6 schematically summarizes the most relevant pathological alterations identified so far in collagen VI-deficient tendon.

By clarifying tendon-specific pathogenic pathways in COL6-RM, this work aims to generate new insights that support the identification of druggable targets and the advancement of novel experimental and clinical interventions. Emphasizing these future directions can foster a sense of hope and engagement among researchers and clinicians committed to improving patient outcomes.

## Data Availability

The original contributions presented in this study are included in the article. Further inquiries can be directed to the corresponding authors.
